# Antiseptic Surgery

**Published:** 1879-10

**Authors:** C. Briggs

**Affiliations:** Nashville, Tenn.


### ANTISEPTIC SURGERY.

By C. [illegible]. BRIGGS, M.D., NASHVILLE, TENN.

*Read Before the Nashville Academy of Medicine.*

In this paper we do not propose to give an extended history of this almost inexhaustible subject, nor shall we attempt, in the limited scope of this essay, to discuss the germ theory upon which the antiseptic system of surgery is based. We shall feel fully satisfied shall we be able to bring before our hearers a clear exposition of the origin, the past career, and the present status of antiseptic surgery.

Antiseptic surgery has reference to dealing with surgical diseases in such a manner as to "prevent the introduction of putrifactive influences in wounds." By its use we seek to promote the healthy repair of wounds, and thereby to prevent them from becoming sources from which are drawn the seeds of such fatal diseases as pyæmia, erysipelas and septicæmia.

To secure the union of wounds, by primary adhesion, has always been the surgeon's greatest ambition.

It is well known that to obtain such a desired end it is necessary: 1st, That the patient's system be in a condition to furnish the proper kind of reparative material; 2d, That anything that may act as a foreign body be rigorously excluded; 3d, That efficient measures be taken to secure free drainage of such fluids as may become injurious; 4th, That the edges of the wound be accurately adjusted; 5th, That absolute, or as near absolute as possible, rest be maintained.

Common experience teaches us that occasionally wounds so treated heal with a most gratifying rapidity, but even with the greatest care and skill, ordinary wounds often take an entirely different course. It may be due to a neglect of one of these necessary conditions, or the refusal to heal depends upon a vitiated state of the patient's health. At any rate, an inflammation over and beyond that necessary for the production of reparative material is induced. As a consequence, we meet with an abundant discharge that distends the wound, which, unless provided for by the judicious employment of drainage, adds to the already excited condition of the parts. In addition to this disturbing element of the healing forces, we have another, even more powerful, in the entrance of the air into the wound, and its power to induce putrefactive changes in fluids of the wound. From time immemorial, it has been a universally recognized fact that atmospheric air exercises a peculiarly noxious effect upon the process of repair in wounds. What there is resident in the air that so powerfully affects open wounds, is a single question to which there have been innumerable answers. That such a fact is true, we see in the difference of the mode of healing between open and subcutaneous wounds; in the latter, protected against the entrance of air, healing is rapid and unattended with any of the dangers of the former. Upon this well known truth rests the many methods of opening chronic abscesses, the entrance of air into which is attended with such injurious consequences.

Thus, pus of a chronic abscess, previous to evacuation by incision, is perfectly free from odor. If opened by free incision, and the air be allowed to enter its cavity, the pyogenic membrane is stimulated into renewed activity, the pus rapidly accumulates, and though absolutely free from odor before evacuation, it has now acquired the distinctive fetor of putrefactive decomposition.

The entrance of air into wounds and abscesses induces certain putrefactive changes that operate, not only to prevent the healing process, but too frequently to lead to the fatal attendants of blood poisoning by absorption from putrescent materials. The industry and ingenuity of Pasteur has led to the discovery of the important fact that the air contains certain spores or germs that induce putrefaction in fluids and solids, just as the yeast plant produces the alcoholic fermentation in a saccharine solution. Tyndall corroborated the statements of Pasteur, and by a series of practical experiments proved that the dust of the atmosphere was pregnant with germs that occasion putrefaction. The illustration of the presence of these germs is familiar to all. Let a sunbeam into a dark room, and these motes are seen in infinite number floating in the beam. In 1867, Mr. Joseph Lister, of Edinburgh, reasoning from the experiments of Pasteur and Tyndall that putrefaction and suppuration were similar processes due to the presence of these atmospheric organisms, instituted that method of treating wounds, now commonly known as the antiseptic system.

The idea involved in all antiseptics applied to the treatment of wounds is to antagonize these disease germs, and thus exclude them entirely from wounds. Any agent, therefore, that is fatal to these germs, may be termed an antiseptic.

Heat is known to be destructive to them, as is evidenced in the preservation of fruits by subjecting them to boiling. Cold is also an antiseptic. Esmarch applied this principle to the treatment of gunshot wounds, and Billroth speaks highly of its use as an antiseptic. Lemoin, several years before Lister announced his carbolic acid treatment, employed a powder made of coal-tar and lime in the treatment of wounds, and his practice was extensively adopted by both French and German surgeons. Boracic acid is an admirable antiseptic, and is still frequently employed. It is said to be inferior to carbolic acid in being non-volatile, and therefore cannot be made to penetrate the innermost recesses of the wounds. Chloride of zinc and the metallic salts, are, under certain circumstances, useful as antiseptics. Salicylic acid and thymol are frequently used as antiseptics, but do not possess the same reputation as carbolic acid. Mons. Alphonse Guerin uses successive layers of cotton-wool in the treatment of wounds, on the principle that it acts as a filter and strains the air of its impurities. He claims flattering results from this dressing.

Dr. Callender uses a combination, it may be said, of the cotton-wool and carbolic acid methods, paying particular attention to drainage, and his results, after capital operations, have never been equalled.

Dr. Wood, of New York, treats wounds by the open method; but frequently washes the parts with carbolic acid lotions. Mons. Ollier uses what he terms the "occlusion immovable" method, in which, after applying the cotton-wool dressing of Guerin, he envelopes the whole with a silicate bandage.

Of all the methods, of which we have only named the most noticeable, Mr. Lister's is most remarkable for its attention to details. One writer has remarked, that to be successful, one must act as if the germs were to be seen on everything.

No half-way measure will suffice ; every detail must be scrupulously observed.

Like all innovations on the old style of things, this method of treating wounds has met with the most determined opposition from all quarters. Fischer has found the bacteria beneath the antiseptic dressings of Lister, but the latter declares that they belong to a different species from the organisms that exert putrefactive power over wound discharges. Ranke and Von Bisrch-Hirschfield also found bacteria septica in antiseptic dressings, but in every case the wounds pursued the favorable course that generally follows Lister's plans.

Again, opponents of the doctrine contended that the same bacteria are found in abscesses when the skin is not broken, that therefore they must exist in the blood itself, and why do they not produce the same toxic effect as when introduced from without. Lister says that their presence in abscesses is rare, but refuses to believe that every foul smelling collection of pus has undergone putrefective changes. That these germs do not induce putrefective changes from within, is evidenced by the course pursued by simple fracture.

Dr. Perren, in the *Union Medicale*, contends that alcohol is more suitable than carbolic acid, on the ground that two things are to be taken in view in the consideration of putrefactive changes in wounds, the germ and the soil. Carbolic acid, while it may destroy the germs, does not affect the condition of the wound as alcohol does. Alcohol renders albuminous matters imputrescible, checks hemorrhage and has coagulating power, and can penetrate the tissues of the body.

Prof. Wood, in *Medical Times and Gazette*, opposed the method on the ground that in hospitals, when pyæmia, erysipelas, etc., was raging, it did not insure the patient's immuity, though it did prevent the bad odors in wounds that appeared in those treated otherwise.

On this side of the Atlantic it is rapidly growing in favor. Even the most determined enemies of the doctrine are forced to admit that the results obtained from it are incomparable. As surgeons grow more familiar with the method, and more careful with the long train of details, they become more and more convinced that Lister's antiseptic method is not a failure. Prof. Hodgen, of St. Louis, in an address before the International Medical Congress, says, that while it or any other antiseptic may fail to prevent the entrance of bacteria into wounds, "by its employment the rapidity of putrefactive changes may be diminished the absorption of septic material limited, and the eliminative functions stimulated so that septicæmia may not occur."

Dr. D. H. Agnew, in an address before the Pennsylvania State Society says of it, "the number and variety of cases treated during the past six months, when compared with a similar number and similar variety (of wounds) treated after ordinary plans, have yielded results so greatly superior, that I have no doubt in my mind we are on the eve of an entire change in the surgical dressing of wounds." It is needless to multiply the opinions of the leading surgeons in this country, suffice it to say, that the current of opinion is in favor of the method. Let us compare the surgery of to-day with that of but a few decades since, and we can not but be favorably impressed with the impetus given to surgery. A compound fracture which then was almost invariably consigned to the amputating knife, is now, with the protective influences of the antiseptic plan, made to heal as certainly and readily as the simple fracture. Contused and lacerated wounds of large extent then formed the most dreaded accidents in surgery, especially when treated in hospitals, for they were continually threatened with pyæmia, septicæmia and erysipelas, diseases which have now almost become novelties. To open a chronic abscess then, was to invite death to take the patient; with the aid of the antiseptic plan they may now be fully evacuated without the least resulting harm. By its aid, surgeons have dared operations which, but a few years since, would have been termed murderous. The abdomen is fearlessly opened, the gall bladder incised, the spleen removed, and the chest cavity entered. The mortality of all capital operations has lessened since its introduction. In short, we regard the antiseptic method of treating wounds, as one of the greatest boons ever given to surgery.

To carry out the antiseptic system, there is needed a steam atomizer in which a solution of 1-30 of carbolic acid is to be atomized; protection, consisting of carbolized oiled silk; antiseptic gauze, prepared by saturating coarse muslin with a mixture of 1 part carbolic acid, 5 parts paraffine and 7 parts resin; carbolized catgut ligatures, which are cut short and left in the wound; carbolized rubber drainage tube. The carbolized spray is made to play on the surgeon's hands and the site of the operation. The part operated on should be shaved of its hairs and carefully washed with 1-20 sol. carb. acid. The wound is approximated with silver sutures. the protective made to cover the wound and the surface several inches on every side; the gauz is applied in 8 thicknesses, the mackintosh cloth being inserted between the two upper layers of the same, and the whole covered with carbolized bandages. The wound is dressed with the same antiseptic precautions the next day, and afterwards allowed to remain until the appearance of discharge through the dressings. The presence of pus is not an evidence of the failure of the dressing, and should not deter the surgeon from persisting in the treatment.

However, many cases proceed to recovery without the formation of one drop of pus, as in a case in our practice, in which the femoral artery was ligatured under the antiseptic method in all its details. The antiseptic spray is gradually falling into disfavor, and doubtless will ere long be abandoned. A great objection to its use is the interference of the mist with sight of the minute points to be observed. Special care should be taken to have the solutions of the proper strength, as stronger solutions acting as irritants, excite too much inflammation.

After consideration of the points we have attempted to make, we may draw up the following conclusions :

1. That, although, the germ theory of Pasteur has not yet received full confirmation, the principle as applied to the treatment of wounds is correct.

2. That of the numerous antiseptic methods now in use, either Lister's carbolic acid dresing or Guerin's cotton-wool offers the most rational plan of treating wounds according to the principle deducted from Pasteur's germ theory.

3. That until the expense of apparatus, and the tedious detail of Lister's method is lessened and the dressing simplified, it will never become generally popular.

4. That the unprecedented success of antiseptics in the treatment of wounds challenges the admiration of its most determined enemies, and announces a new era in surgery.—*Nashville Journal of Medicine and Surgery.*

